# Whole-Genome Sequence of *Escherichia coli* Serotype O157:H7 Strain PA20

**DOI:** 10.1128/genomeA.01460-16

**Published:** 2017-01-12

**Authors:** Gaylen A. Uhlich, George C. Paoli, Xinmin Zhang, Edward G. Dudley, Hillary M. Figler, Bryan J. Cottrell, Elisa Andreozzi

**Affiliations:** a Molecular Characterization of Foodborne Pathogens Research Unit, USDA, Agriculture Research Service, Eastern Regional Research Center, Wyndmoor, Pennsylvania, USA; b BioInfoRx, Inc., Madison, Wisconsin, USA; c Department of Food Science, Pennsylvania State University, University Park, Pennsylvania, USA; d The Huck Institute of Life Sciences, Pennsylvania State University, University Park, Pennsylvania

## Abstract

*Escherichia coli* serotype O157:H7 strain PA20 is a Pennsylvania Department of Health clinical isolate. It has been used to study biofilm formation in O157:H7 clinical isolates, where the high incidence of prophage insertions in the *mlrA* transcription factor disrupts traditional *csgD* biofilm regulation. Here, we report the complete PA20 genome sequence.

## GENOME ANNOUNCEMENT

Shiga toxin–producing *Escherichia coli* causes hemorrhagic colitis, which may progress to severe sequelae such as hemolytic uremic syndrome. In the United States, O157:H7 is the most important serotype, in numbers of both sporadic cases and large outbreaks. *E. coli* subjected to low nutrient and stress conditions is conferred protective advantages by forming biofilms, a process controlled by the central transcriptional regulator CsgD (1). However, studies have shown that biofilm formation is reduced or absent in >95% of O157:H7 clinical samples, a consequence of prophage insertions in the *mlrA* transcription factor required for maximum RpoS-dependent *csgD* expression (2, 3). Prophage-bearing strains restored to stronger *csgD* expression and biofilm formation through various genetic modifications have been described, but many remain uncharacterized (4). One *E. coli* O157:H7 strain used extensively in these studies was strain PA20. Here, we report the complete sequence of strain PA20 for use as a reference for DNA comparisons and RNA mapping with previously identified biofilm-forming variants.

Strain PA20 is a clinical isolate from the Pennsylvania Department of Health, Exton, Pennsylvania, USA. DNA was extracted from a frozen cell pellet of PA20 using the Qiagen Genomic-tip 100/G kit (Qiagen, Valencia, CA, USA). The gDNA was sequenced, and sequences were assembled at the University of Delaware Sequencing and Genotyping Center using the PacBio RS II SMRT DNA sequencing system and HGAP Assembly.3 software (Pacific Biosciences, Menlo Park, CA, USA). Two PacBio sequencing runs generated slightly different assemblies, each containing six contigs, which were aligned to the EDL 933 (NZ_CP008957) and Sakai (NC_002695) genomes using Mauve (http://darlinglab.org/mauve/mauve.html). Contigs were ordered and connected to form single chromosomal and plasmid sequences. Finally, duplicated assembly end-sequences were removed to circularize the sequence and the start and end of the genome sequence was chosen to match the GenBank records for EDL 933 and Sakai.

Total DNA was also sequenced using Illumina MiSeq (ProteinCT, Madison, WI, USA). Approximately 4.5 million, 2 × 250-bp paired-end reads from a PA20 Nextera DNA library (Illumina) were evaluated by FastQC. Adapters and low-quality sequences (<Q20) were removed using Trimmomatic (http://USADELLAB.org, Aachen, Germany). The Illumina MiSeq reads were aligned to the PacBio assemblies using Burrows–Wheeler Aligner (http://bio-bwa.sourceforge.net) and two single-base sequencing errors, likely PacBio in origin, were corrected.

The PA20 genome contained a single 5,525,846-nucleotide (nt) chromosome and one plasmid of 92,755 nt. The genome was annotated by the NCBI Prokaryotic Genome Annotation Pipeline (https://www.ncbi.nlm.nih.gov/genome/annotation_prok), and putative prophage locations were predicted using PHAST (5) and comparison with the Sakai genome. Although the genome content was similar to that of Sakai, the PA20 draft assembly contained two large genome inversions and several smaller rearrangements. The largest inversion (>1,400 kb) mapped between prophage sequences located in Sp4 and Sp14 (Sakai annotation), while a second inversion (>400 kb) within the largest inversion had termini in prophage Sp9 and Sp12. Due to extensive sequence redundancy in the flanking regions of the inverted segments, alternative assemblies of that region cannot be ruled out using the existing data.

### Accession number(s).

This whole-genome shotgun project has been deposited at DDBJ/EMBL/GenBank under the accession numbers CP017669 (genome) and CP017670 (plasmid).
